# Treatment Outcome of Severe Malaria and Associated Factors among Adults Admitted in Arba Minch General Hospital, Southern Nation Nationality and People's Region, Ethiopia

**DOI:** 10.1155/2021/6664070

**Published:** 2021-04-12

**Authors:** Solomon K. Bekele, Muluken B. Ayele, Asmare G. Mihiret, Negalign G. Dinegde, Hussen Mekonen, Gesila E. Yesera

**Affiliations:** ^1^Arba Minch University College of Medicine and Health Science, Ethiopia; ^2^Addis Ababa University College of Health Sciences, Ethiopia

## Abstract

**Introduction:**

Malaria is a protozoan disease transmitted by the bite of infected female *Anopheles* mosquitoes. Progression to severe and fatal disease is largely but not entirely confined to *Plasmodium falciparum* infections. Malaria is a major public health problem in Ethiopia despite relatively low malaria prevalence compared to most other malaria-endemic countries in Africa. In Ethiopia, a nationwide report during 2015 showed that the total number of deaths associated with malaria was 1561.

**Methods:**

A retrospective cross-sectional study was conducted in Arba Minch General Hospital on February 2019. Data were collected from a patient record who was admitted with severe malaria in the past four years from Sept. 2015 to Aug. 2018.

**Results:**

This study included a total of 387 patients with severe malaria. The mortality rate associated with severe malaria in the year between 2015 and 2018 at Arba Minch General Hospital was 5.7%. Comorbidity, impaired consciousness, and acidosis were significantly associated with mortality, at significant level of *P* < 0.05.

**Conclusions:**

Comorbidity, impaired consciousness, and acidosis were found to be poor prognostic indicators for patients with severe malaria.

## 1. Introduction

Malaria is a protozoan disease transmitted by the bite of infected female Anopheles mosquitoes [1, 2]. Progression to severe and fatal disease is largely but not entirely confined to *P. falciparum* infections. Although they contribute much less than *Plasmodium falciparum* to the global burden of severe malaria, both *Plasmodium vivax* and *Plasmodium knowlesi* can also cause severe disease and they do kill [3].

According to the WHO criteria published in 2014, severe malaria is defined as one or more of the following, occurring in the absence of an identified alternative cause, and in the presence of *P. falciparum* asexual parasitaemia [3]:

1. Impaired consciousness: a Glasgow Coma Score < 11 in adults
2. Acidosis: severe acidosis manifests clinically as respiratory distress—rapid, deep, and laboured breathing
3. Hypoglycaemia: blood or plasma glucose < 2.2 mM (<40 mg/dl)
4. Severe malarial anaemia: a haemoglobin concentration < 5 g/dl or a haematocrit of <15%
5. Prostration/extreme weakness: inability to walk or sit up without assistance. This is the milder and benign end of the neurological manifestations spectrum, potentially dangerous unless treated urgently
6. Renal impairment (acute kidney injury): plasma or serum creatinine > 265 *μ*M (3 mg/dl) or blood urea > 20 mM
7. Jaundice: plasma or serum bilirubin > 3 mg/dl
8. Pulmonary oedema: radiologically confirmed, or oxygen saturation < 92% on room air with a respiratory rate > 30/min, often with chest indrawing and crepitations on auscultation
9. Significant bleeding: including recurrent or prolonged bleeding from nose gums or venipuncture sites; haematemesis or melaena
10. Shock: compensated shock is defined as capillary refill ≥ 3 s or temperature gradient on leg (mid to proximal limb), but no hypotension
11. Hyperparasitaemia*: P. falciparum*parasitaemia > 10%

In 2017, an estimated, 219 million cases of malaria occurred. Even though there was an estimated 20 million fewer malaria cases in 2017 than in 2010, data for the period 2015–2017 highlight that no significant progress in reducing global malaria cases was made in this timeframe [4].

A multicenter trial conducted in Bangladesh, Myanmar, India, and Indonesia from June 2003 to May 2005 on the relationship between age and mortality associated with severe malaria revealed that mortality increased stepwise, from 6.1% in children (age, <10 years) to 36.5% in patients aged >50 years. Compared with adults aged 21–50 years, the decreased risk of death among children and the increased risk of death among patients aged >50 years were independent of the variation in presenting manifestations. Coma and metabolic acidosis did not vary with age and were the strongest predictors of a fatal outcome. The number of severity signs at hospital admission also had a strong prognostic value [5].

With use of data from a trial conducted in Southeast Asia (*n* = 868) to predict the outcome of severe malaria in adults, acidosis (base deficit) and cerebral malaria (measured as Glasgow Coma Score) were the main independent predictors of outcome. This study suggests that patients presented with altered level of consciousness and acidosis had poor prognosis [6].

Although there was remarkable progress in recent years, malaria remains a leading cause of sickness and death across much of sub-Saharan Africa. Majorly malaria impacts the rural poor, typically people who must walk for miles to seek treatment. It is also a leading cause of absenteeism among employees, increased health care spending, decreased productivity, and approximately 50 percent of all preventable school absences in Africa. Malaria helps to trap families in a vicious cycle of disease and poverty [7].

The 2017 WHO age-adjusted death rates estimates associated with severe malaria reported that, in Africa, a total of 43 countries including Ethiopia are under high death rate. Ethiopia accounts 5.43 deaths per 100,000 [8].

Malaria is a major public health problem in Ethiopia despite relatively low malaria prevalence compared to most other malaria-endemic countries in Africa. Unstable malaria transmission patterns make Ethiopia prone to focal and multifocal epidemics that have on occasion caused catastrophic public health emergencies. Malaria is seasonal in most parts of Ethiopia, with variable transmission and prevalence patterns affected by the large diversity in altitude, rainfall, and population movement [9].

In 2014/2015, the total number of laboratory-confirmed plus clinical malaria cases in Ethiopia was 2,174,707 [10]. Another study conducted on mortality rates of malaria in Ethiopia during 2015 showed the total number of deaths associated with malaria was 1561 which is a significant number suggesting an attempt should be done to bring down deaths [11]. So, this shows that malaria is a problem on Ethiopia.

This study is aimed at identifying the treatment outcome of patients with severe malaria and associated factors which will help to contribute on the prevention and management of severe malaria; the study will also help to set plan on the future program of severe malaria treatment. Further, it will be used as a baseline for other studies.

Generally, areas located less than 2,000 meters above sea level (<2,000 m) in altitude are considered malarious areas [12]. The study area (Arba Minch) is at an elevation of 1,285 meters ASL [13],which makes it to be malarious area, and there are frequently reported cases of malaria.

## 2. Materials and Methods

### 2.1. Study Area and Period

The study was conducted at Arba Minch General Hospital. It is a general hospital located in Arba Minch town, Gamo Gofa zone, Southern Nation Nationalities and Peoples Region, 505 km, south of the capital Addis Ababa with an elevation of 1285 meters ASL. Arba Minch General Hospital officially commenced its functioning in 1963 and has currently been delivering its health care services in the medical, surgical, gynecological, and pediatrics wards, for more than 1,000,000 populations.

### 2.2. Study Design

Institution-based retrospective cross-sectional study was used.

### 2.3. Sample Size

Sample size was calculated using a single population proportion sample size determination method by assuming 50% of case fatality to yield the maximum representative sample size with 95% confidence interval.

(1) $$\begin{array}{c} \frac{n={\left(Z_{\alpha /2}\right)}^{2}*p\;\left(1-p\right)}{d^{2}}, \end{array}$$

where *n* is the required sample size; *z* is the critical value at 95% CI (1.96); *p* is the prevalence rate; since the magnitude of mortality is not known, p is taken as 50% i.e., 0.5; and margin of error (*d*) to be 5% (*d* = 0.05).

(2) $$\begin{array}{c} \frac{n={\left(1.96\right)}^{2}*0.5\;\left(1-0.5\right)}{{\left(0.05\right)}^{2}},\;n=384. \end{array}$$

Adding 10% contingency for incomplete medical record to 384 equals 422.

### 2.4. Sampling Technique

The records and charts of patients with severe malaria over the past four-year period, from September 2015 to August 2018, was retrieved from the record room by taking medical record number from adult medical ward patient admission and discharge registration log book.

### 2.5. Diagnostic Test

In order to test for the presence of *Plasmodium* species, direct microscopy was used for all patients. Thin and thick blood films were prepared in order to test for the type of species and parasite load, respectively. Blood sample is collected from the fingertip using a sharp-pointed needle.

### 2.6. Ethical Consideration

Ethical approval was granted from Research Ethics Committee of Addis Ababa University Collage of Health Science school of Nursing and Midwifery, as this was a retrospective study individual patient informed consent was not required.

### 2.7. Data Processing and Analysis

Data were entered to EpiData- version 3.1. and then exported to SPSS version 25 for analysis. Descriptive statistic was performed to summarize results using tables. In bivariate logistic regression analysis; variables having value less than 0.25 was entered in to multivariate logistic regression to assess statistical association between the outcome variable and independent variables; odds ratio was calculated to measure the magnitude of association using 95% confidence interval (CI) and *P* value (<0.05).

## 3. Result

### 3.1. Sociodemographic Characteristics of Study Participants

In this study, 387 patients with severe malaria were included. From the total of 387 patients with severe malaria in the year between September 2015 to August 2018, 231 (59.7%) and 156 (40.3%) were male and female, respectively. The mean age was 26.16 (SD ±9.9), majority of respondents 289 (74.7%) lie in the age group 18-27 years. Almost many 361 (93.3%) of patients with severe malaria came from area of high transmission and 26 (6.7%) from a low transmission area.  Table 1 below shows details of sociodemographic variables.

### 3.2. Clinical and Other Related Factors

As indicated in Table 2, out of 387 cases, 357 (92.2%) patients had come to hospital in ≤5 days since the onset of sign and symptoms. From 387 cases of severe malaria, 214 (55.3%) had single WHO defined severity indicator and the remaining 173 (44.7%) had 2 or more overlapping severity indicator during admission (Table 2).

### 3.3. Patient's Status of Comorbidity

As described in Table 3, out of 387 patients with severe malaria, 344 (88.9%) patients have no comorbidity (Table 3). From a total of 43 patients with comorbidity, 37 (86%) had acute illness and 6 (14%) had chronic illness (Table 3).

### 3.4. Patient Manifestation during Admission

From 387 cases of severe malaria (Table 4), 214 (55.3%) had single WHO defined severity indicator and the remaining 173 (44.7%) had two or more overlapping severity indicator during admission (Table 4).

### 3.5. Treatment Outcome, Manifestation Involved, and Mortality in Severe Malaria

Of all 387 patients with severe malaria, 365 (94.3%) had recovered and 22 (5.7%) died. The overall case fatality rate was 5.7% (22/387). From 22 deaths registered, 11 had comorbidity (five patients had acute gastro enteritis, two patients had acute kidney injury, two patients had retroviral infection, and two patients each had urinary tract infection and hypertension). The gender-specific case fatality was 5.1% (12/231) in males and 6.4% (10/156) in females. Out of 22 deaths, 9 patients had both impaired consciousness and acidosis (Table 5).

### 3.6. Death Risk Factor Associated with Severe Malaria

As mentioned in Table 6, impaired consciousness, acidosis, and comorbidity found to be significantly associated with death related to severe malaria, *P* value less than 0.05.

Cases that have impaired consciousness were 4.175 times more likely to die than those who did not have impaired consciousness (CI = 1.265 - 13.782, AOR = 4.175). Cases that have acidosis were 5.735 times more likely to die than those who did not (CI = 1.265 - 13.782, AOR = 5.735). Cases that had a co-morbidity were 13.163 times more likely to die than those who did not have comorbidity (CI = 4.168-41.575, AOR = 13.163) (Table 6).

## 4. Discussion

In Ethiopia, clinical malaria incidence rate has dropped from an average of 43.1 cases per 1000 population annually between 2001 and 2010 to 29 cases per 1000 population between 2011 and 2016. Death associated with malaria has declined from 2.1 deaths per 100,000 population between 2001 and 2010 to 1.1 deaths per 100,000 population between 2011 and 2016. That was due to the new policy of free malaria prevention and treatment, which addresses the poor whom are more prone to the infection [14].

There is no recent study conducted in Ethiopia on treatment outcome of severe malaria among adults. Thus, this study had tried to assess treatment outcome of severe malaria among adults admitted in Arba Minch General Hospital during September 2015 to August 2018, Southern Ethiopia. It is necessary to identify factors influencing treatment outcome of severe malaria and reduce the risk of mortality associated with the disease.

The present study included 387 patients with severe malaria and revealed that 59.7% of cases were male and 40.3% were female with higher male to female ratio of 1.48 : 1 which is nearly similar to a study conducted in India Maharashtra which shows a male to female ratio of 2.23 : 1 [15], unlike with a study conducted in Gondar referral hospital, with male to female ratio of 0.83 : 1 [16]. This difference in the magnitude of severe malaria between male and female could be due to the difference in the sociocultural status of the two study areas.

In this study, adult population age group between 18 and 27 (74.7%) are more presented with severe malaria, similar to a study conducted in Gondar Referral Hospital which is higher among age group 20-29 (36.5%) and in India Maharashtra which is again higher among age group 20-29 (34.55%) [15, 16]. This occurred most likely because of an active working age group which makes them vulnerable to infection.

Out of 387 cases, 214 (55.3%) cases had a single severity indicator and 173 (44.7%) cases had two or more overlapping severity indicators. Taking into consideration the overlapping features of severe malaria presenting sign and symptoms, prostration/extreme weakness was seen in the majority of cases accounted 38.3% and hyperparasitaemia seen among 30.5% of the cases. This is consistent with the findings of a study conducted in Malawi Blantyre Queen Elisabeth Hospital [17]. Other presenting features in decreasing order were severe malarial anemia, impaired consciousness, and acidosis.

Regarding the malarial species, *P. falciparum* predominates which accounted for 90.4% of the cases and *P. vivax* accounted 7.1% of the cases which is comparable to the study conducted in Gondar Referral Hospital [16].

In this study, the overall mortality rate associated with severe malaria was 5.7% (22/387) which is lower than similar study conducted in Gondar Referral Hospital, Northern Ethiopia, and a prospective observational study conducted Maharashtra, India, which was 28.4% and 13.63%, respectively [15, 16]. This difference in mortality shows an improvement on the treatment regimen possibly the newly used Artemisinin derivative (artesunate) antimalarial treatment [18].

Case fatality rate was highest among patients with comorbidity (25.6%) than late comers lasting >5 days (23.3%), impaired consciousness (22.2%), acidosis (20%), and shock (15.7%). In the current study, impaired consciousness and acidosis were two of poor prognostic indicators, which was consistent with the study conducted in Gondar Referral Hospital [16]. Comorbidity is another poor prognostic indicator which was again consistent with findings of a study conducted in western Thailand [19].

## 5. Conclusion

This study showed that severe malaria is one of a major burden of Arba Minch General Hospital. Except for pulmonary oedema and convulsion, many of the WHO severity indicators were observed among cases admitted during 2007-2010 EC. The overall mortality rate associated with severe malaria was 5.7%. Comorbidity, impaired consciousness (GCS < 11), and acidosis were a major determinant of treatment outcome (death).

## Figures and Tables

**Table 1:** Sociodemographic characteristics of patients with severe malaria admitted in Arba Minch General Hospital from September 2015 to August 2018, *n* = 387.

Variable	Category	Frequency	%
Age	18-27	289	74.7
	28-37	36	9.3
	38-47	43	11.1
	48-57	11	2.8
	≥58	8	2.1
	Total	387	100.0
Sex	Male	231	59.7
	Female	156	40.3
	Total	387	100.0
Place of residency based on transmission rate	Low transmission (altitude > 2000 m ASL)	26	6.7
	High transmission (altitude < 2000 m ASL)	361	93.3
	Total	387	100.0

**Table 2:** Clinical, laboratory, and other related factors of severe malaria in Arba Minch General Hospital from September 2015 to August 2018, *n* = 387.

Variable	Category	Frequency	%
Time before admission	≤ 5 days	357	92.2
	> 5 days	30	7.8
	Total	387	100.0
Treatment taken	Quinine	53	13.7
	Artesunate	334	86.3
	Total	387	100.0
Site of drug administration	Intravenous	384	99.2
	Intramuscular	3	.8
	Total	387	100.0
Is there any other associated disease	No	344	88.9
	Yes	43	11.1
	Total	387	100.0
Type of malarial species	Falciparum	350	90.4
	Vivax	30	7.8
	Both	7	1.8
	Total	387	100.0

**Table 3:** Patient's status of comorbidity with severe malaria in Arba Minch General Hospital from September 2015 to August 2018, *n* = 387.

Patient's status of comorbidity	Frequency	Percent
No comorbidity	344	88.9
Acute gastro enteritis^∗^	21	5.4
Acute kidney disease^∗^	11	2.8
Urinary tract infection^∗^	5	1.3
Retroviral infection	4	1.0
Hypertension	2	0.5
Total	387	100

^∗^Indicates acute comorbidity.

**Table 4:** Patient manifestation for severe malaria during admission in Arba Minch General Hospital from September 2015 to August 2018, *n* = 387.

Manifestation	Alone/single manifestation (no. of cases)	Two or more manifestations (no. of cases)	Total no.
Impaired consciousness	16	29	45
Acidosis	—	25	25
Hypoglycaemia	4	14	18
Severe malarial anaemia	8	41	49
Prostration/extreme weakness	115	117	232
Renal impairment	3	21	24
Jaundice	—	3	3
Pulmonary Oedema	—	—	—
Significant bleeding	3	3	6
Shock	5	14	19
Hyperparasitaemia > 10%	60	125	185

A single patient can have one or more manifestations.

**Table 5:** Manifestations involved and mortality in severe malaria in Arba Minch General Hospital from September 2015 to August 2018, *n* = 387.

Manifestation	Total no.	Deaths (out of 22)	Case fatality rate (%)
Impaired consciousness	45	10	22.2
Acidosis	25	5	20
Hypoglycaemia	18	2	11.1
Severe malarial anaemia	49	3	6.1
Prostration/extreme weakness	232	10	4.3
Renal impairment	24	1	4.1
Jaundice	3	—	—
Pulmonary Oedema	—	—	—
Significant bleeding	6	—	—
Shock	19	3	15.7
Hyperparasitaemia > 10%	185	6	3.2

A single patient can have one or more manifestations.

**Table 6:** Death risk factor associated with severe malaria among adults admitted in Arba Minch General Hospital from September 2015 to August 2018, *n* = 387.

Variable	Category	COR (95% CI)	AOR (95% CI)
Age ≥ 40	Yes	4.169 (1.655-10.503)	2.776 (0.825-9.339)
	No	1	1
Place of residency based on transmission rate	Low transmission	1	1
	High transmission	3.465 (1.080-11.119)	0.265 (0.058-1.218)
Time before admission >5 days	Yes	6.939 (2.574-18.703)	2.231 (0.643-7.744)
	No	1	1
Impaired consciousness	Yes	7.857 (3.167-19.494)	4.175 (1.265-13.782)^∗^
	No	1	1
Acidosis	Yes	5.074 (1.699-15.155)	5.735 (1.339-24.554)^∗^
	No	1	1
Shock	Yes	3.444 (0.923-12.850)	0.921 (0.159-5.351)
	No	1	1
Hyperparasitaemia	Yes	0.390 (0.149-1.018)	0.370 (0.114-1.204)
	No	1	1
Treatment taken	Artesunate	1	1
	Quinine	2.537 (0.946-6.808)	2.573 (0.741-8.929)
Is there any other associated disease?	Yes	10.406 (4.184-25.880)	13.163 (4.168-41.575)^∗^
	No	1	1

Note: ^∗^indicates significant at *P* value < 0.05. COD: crude odds ratio; AOR: adjusted odds ratio.

## Data Availability

The datasets used and/or analyzed during the current study available from the corresponding author on reasonable request.

## Abbreviations

ARDS: Acute respiratory distress syndrome
AMGH: Arba Minch General Hospital
ASL: Above sea level
CI: Confidence interval
SNNPR: Southern Nation Nationalities and People's Representative.
